# Advances and trends in the application of functional near-infrared spectroscopy for pediatric assessments: a bibliometric analysis

**DOI:** 10.3389/fneur.2024.1459214

**Published:** 2024-09-06

**Authors:** Lin Gao, Qianqi Lin, Dong Tian, Siying Zhu, Xiantao Tai

**Affiliations:** ^1^First Clinical Medical College, Yunnan University of Chinese Medicine, Kunming, China; ^2^Qujing Medical College, Qujing, China; ^3^Second Clinical Medical College, Yunnan University of Chinese Medicine, Kunming, China

**Keywords:** bibliometric analysis, functional near-infrared spectroscopy, pediatrics, VOSviewer, R-bibliometrix

## Abstract

**Objective:**

The objective is to elucidate the collaboration and current research status in the pediatric field of fNIRS using bibliometric analysis, and to discuss future directions.

**Method:**

Bibliometric analysis was conducted on publications related to pediatric fNIRS research published before June 2024 in the Web of Science Core Collection using VOSviewer software and R language.

**Results:**

A total of 761 documents were retrieved, published by 2,686 authors from 893 institutions across 44 countries in 239 journals. The number of publications has significantly increased since 2012. The United States is the country with the highest number of publications, University College London is the institution with the most publications, Lloyd-Fox Sarah is the author with the most publications and significant influence, and “Neurophotonics” is the journal with the most publications. The current hotspots mainly involve using fNIRS to study executive functions and autism spectrum disorders in children.

**Conclusion:**

The study provides useful reference information for researchers by analyzing publication numbers, collaborative networks, publishing journals, and research hotspots. In the future, there should be an emphasis on enhancing interdisciplinary and international collaboration to collectively dedicate efforts toward the advancement of fNIRS technology and the standardization of research.

## 1 Introduction

In recent years, with the development of brain imaging detection technologies, brain function research has increasingly become the focus of attention for clinicians and researchers in the fields of psychiatry and neurology. Functional Near-Infrared Spectroscopy (fNIRS) is a non-invasive brain functional neuroimaging technique that uses the absorption spectra of substances to be detected in the near-infrared light band (650 ~ 900 nm) to determine their concentration and characteristics. The blood supply to the brain responds locally to its functional changes, and fNIRS calculates activation levels and functional connectivity of brain regions by detecting changes in the concentration of oxyhemoglobin in brain tissue (1). fNIRS has evolved from low-channel to high-channel, from single-brain region to multi-brain region, and whole-brain imaging. Traditional fNIRS could only observe changes in cerebral blood flow metabolism in the prefrontal cortex, creating a bottleneck in the study of brain functional areas. However, the emergence of high-channel fNIRS means that changes in cerebral blood flow metabolism over a larger range of brain areas can be observed, breaking the limitations on the study of brain functional areas and allowing fNIRS as a tool for detecting brain functional activity to develop rapidly (2, 3).

Early diagnosis of abnormalities and dysfunctions in children’s brain function development and timely treatment are urgent issues. Brain function imaging detection is an important means of examining abnormalities in children’s brain functions. More precise diagnosis not only requires detection technology with higher temporal and spatial resolution but also better adaptability for poorer compliance and cooperation from child subjects to ensure accurate test results. fNIRS has advantages such as simple operation, convenience, strong anti-interference, good compatibility, and low requirements for the testing environment. It can be conducted in various natural settings such as schools or hospitals, and infants can even be tested in the arms of their parents. It is suitable for researching brain functions in infants or children with developmental disorders and is one of the most promising methods for studying children’s brain functions. It has been widely used in pediatric brain function imaging research (4–6). With the gradual maturity of fNIRS technology in brain function detection applications and the research needs for children’s brain function development and abnormalities, the application of fNIRS technology in pediatric research is increasingly attracting scholarly attention, and publications on this topic are also increasing. Therefore, this study uses bibliometrics analysis to scientifically assess the quantitative and qualitative value of publications on this research topic, describing and summarizing the historical process, current research status, and development trends in this field, providing references for scholars within the domain.

## 2 Materials and methods

### 2.1 Data collection

Using the search terms (“fNIRS” OR “functional near-infrared spectroscopy “) AND (“child” OR “children” OR “pediatrics” OR “pediatric” OR “infant”) to search for literature in the Web of Science Core Collection (WoSCC) database from its inception until May 31, 2024. The search is limited to articles and reviews published in English. The retrieved literature data is downloaded and imported into Noteexpress software, where it is independently checked by two researchers.

### 2.2 Data analysis

This study employs R-bibliometrix and VOSviewer for descriptive statistics and bibliometric analysis, with Scimago Graphica and Pajek aiding in the creation of visual analysis graphs. R-bibliometrix (7) is an open-source tool based on R for bibliometric research. In this study, R-bibliometrix is used to perform descriptive statistics on the number or impact of publications by countries, institutions, authors, and journals, and to analyze hot keywords over the past decade. VOSviewer (8) is software for bibliometric analysis that executes similarity visualization based on information such as publication country, institution, author, and keywords. It creates bibliometric maps or network graphs through co-occurrence analysis (9). This study uses VOSviewer (version 1.6.16) for co-occurrence analysis of countries, institutions, authors, and keywords, and employs Scimago Graphica and Pajek to assist in creating maps and network graphs.

## 3 Results

**Figure 1:**
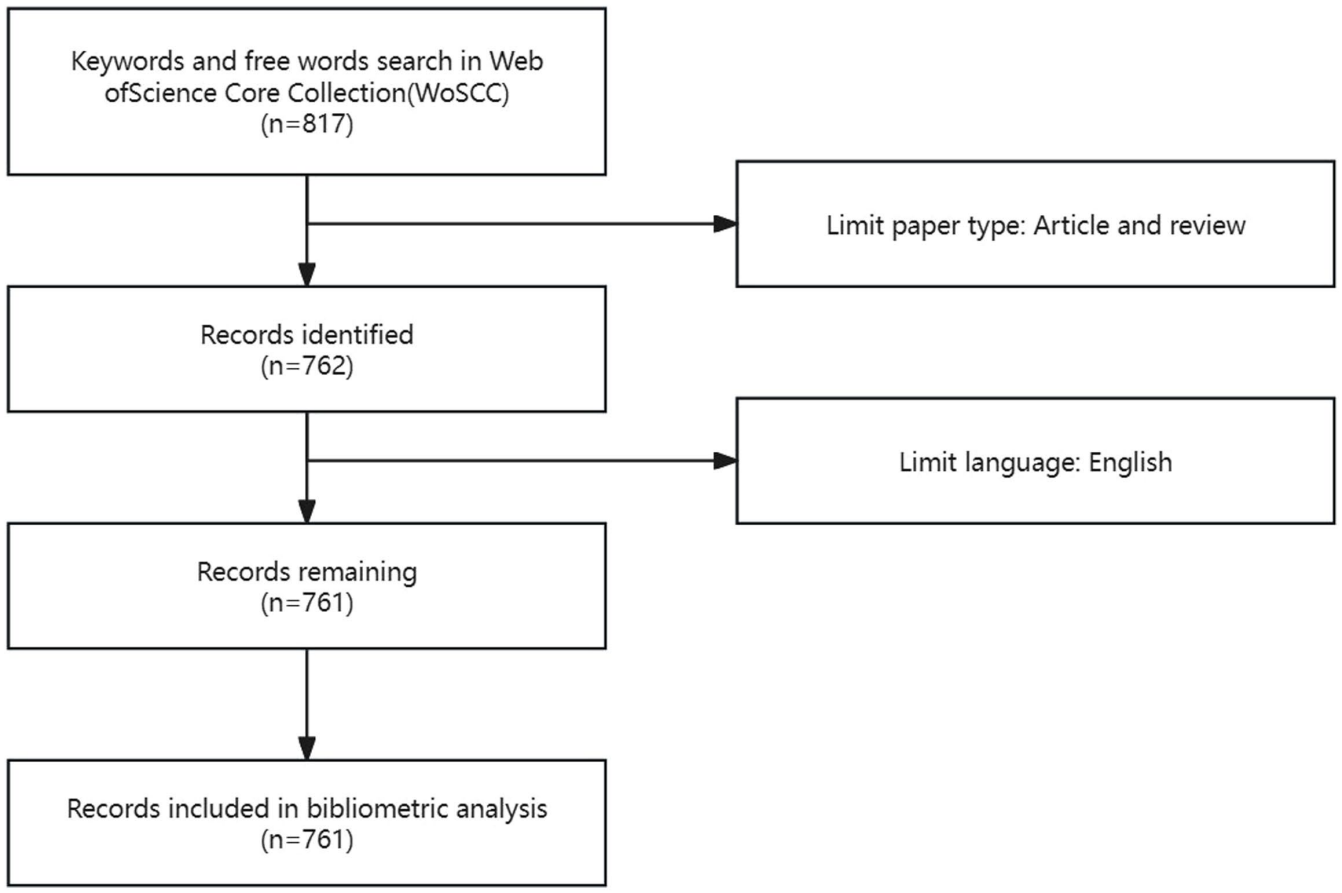
Flowchart of literature search and screening process.

**Figure 2:**
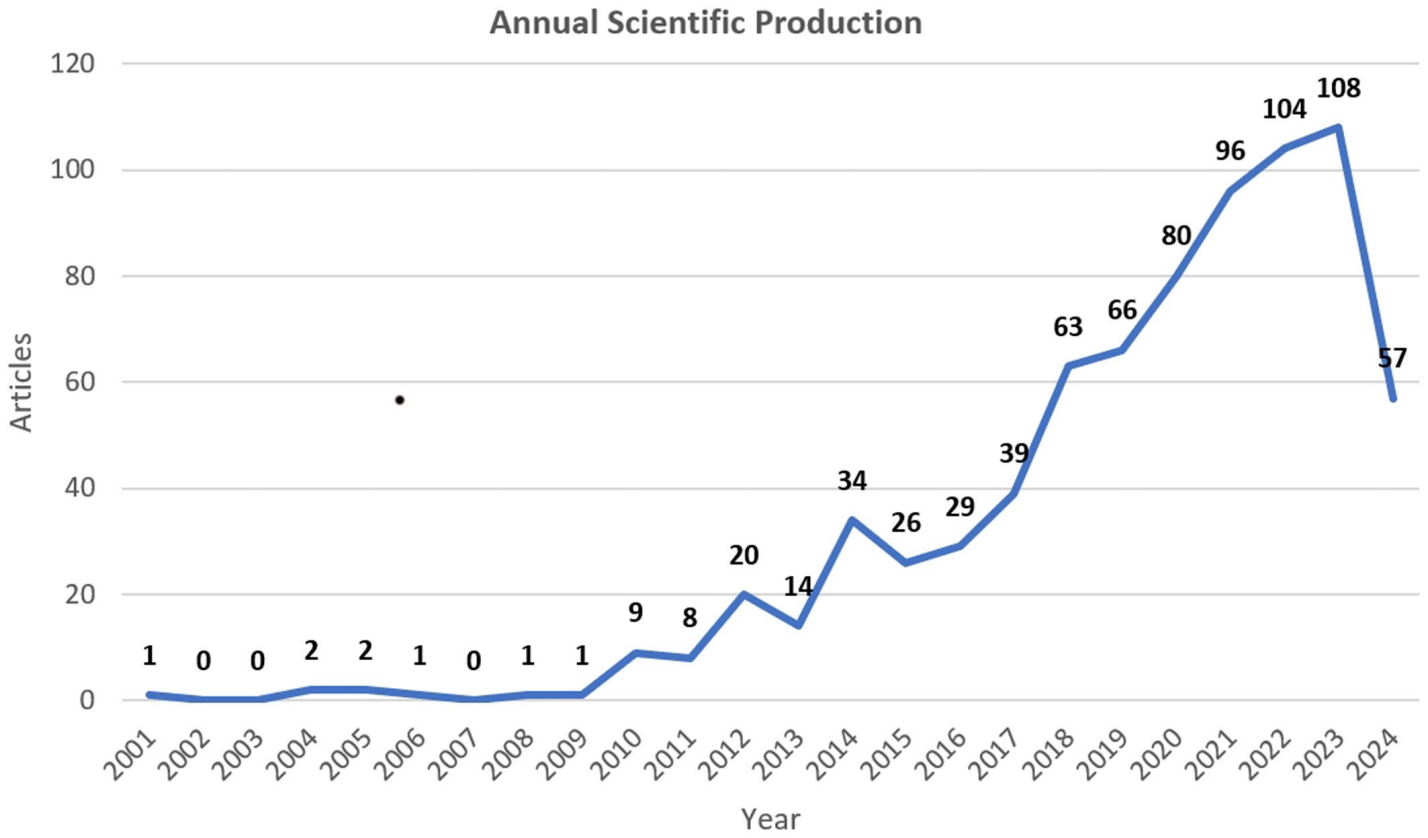
The annual distribution of publications (as of May 31, 2024).

A total of 761 publications were included through retrieval and screening (Figure 1). As shown in Figure 2, research related to fNIRS in pediatrics began in 2001 and has been on an upward trend until 2024. Up to May 31, 2024, the year with the highest number of publications was 2023, with an average of 108 articles published annually, and the output for 2024 has already exceeded half of that in 2023.

### 3.1 Analysis of countries

**Figure 3:**
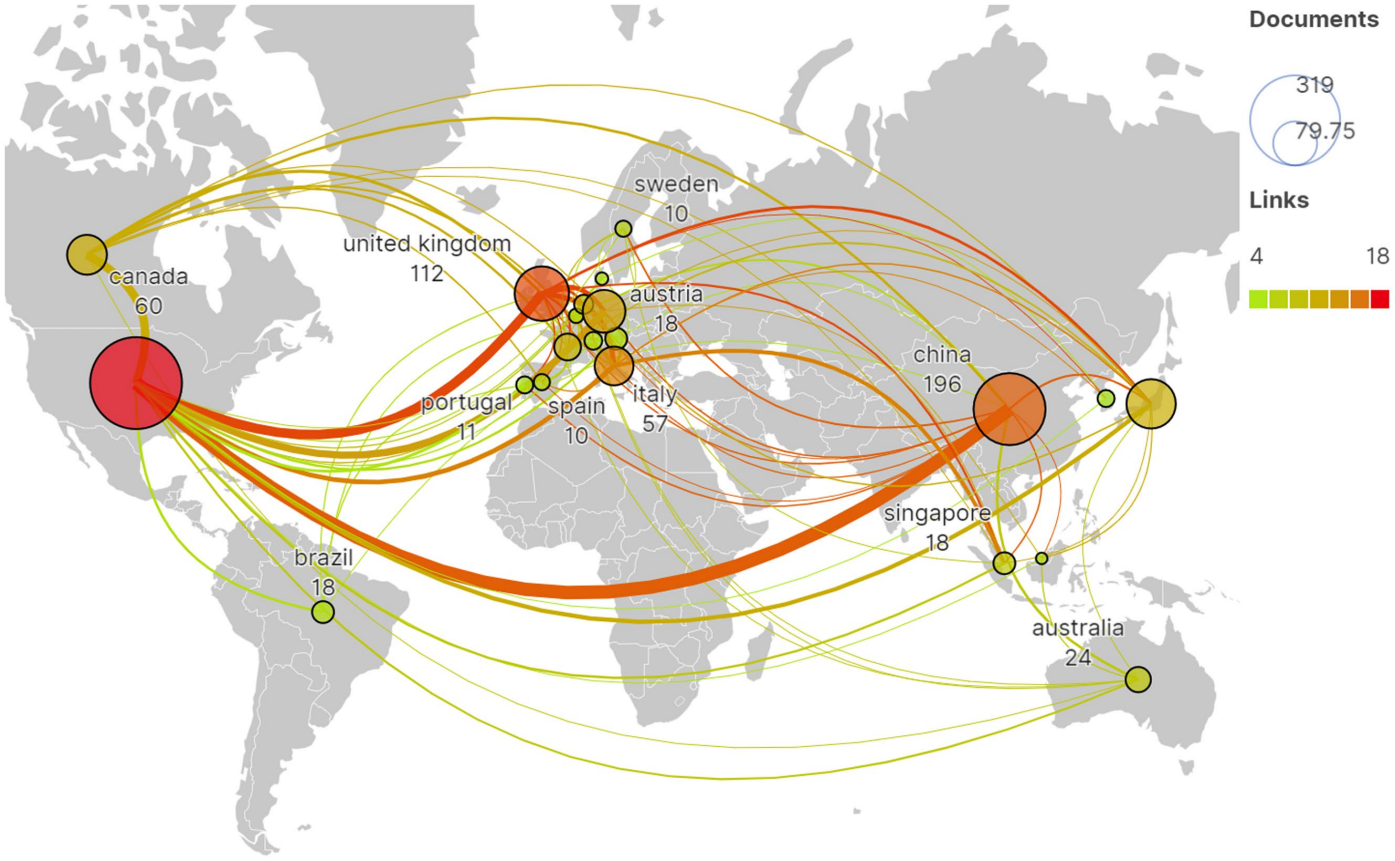
The global distribution of publications and the international cooperation network among countries.

A total of 44 countries have provided publications for the research on fNIRS in pediatrics, with Table 1 showing the top 10 countries in terms of the number of publications. Three countries have published more than 100 papers, among which the United States has the most publications (319/761, 42%), followed by China (196/761, 26%) and the United Kingdom (112/761, 15%). A selection of 21 countries with a publication volume of at least 5 was made for the analysis of international cooperation. Figure 3 demonstrates the active collaboration between these countries, especially the close cooperation between China and the United Kingdom, as well as between China and the US.

**Table 1:** Publication metrics of articles of the top 10 countries by number of publications.

Rank	Country	Documents	Citations
1	United States	319	7,243
2	China	196	2,337
3	England	112	3,359
4	Japan	92	1766
5	Germany	69	2,344
6	Canada	60	1,305
7	Italy	57	2,827
8	France	27	676
9	Australia	24	195
10	Austria	18	331

### 3.2 Analysis of institutions

**Figure 4:**
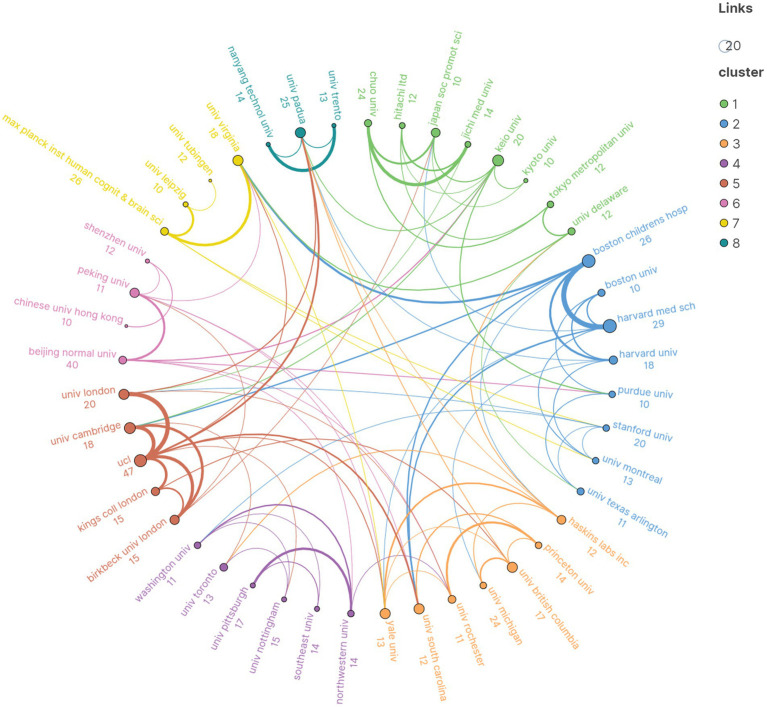
The distribution of institutional publications and the cooperation network among institutions.

A total of 893 institutions participated in the fNIRS pediatric research. Publication statistics and collaborative network analysis were conducted on 45 institutions with a publication count of 10 or more. The results showed that University College London (47/761, 6%) contributed the most publications, followed by Beijing Normal University (40/761, 5%) and Harvard Medical School (29/761, 3%). Clusters 1 and 2 were the two clusters with the highest number of institutions and publications. Cluster 1, consisting of eight institutions including Chou University and Keio University, published 114 articles, while Cluster 2, composed of Boston Children’s Hospital and Harvard Medical School among others, published 137 articles. Additionally, Cluster 5, made up of five institutions including University of London and University College London, published 115 articles despite having fewer institutions (Figure 4).

### 3.3 Analysis of authors

**Figure 5:**
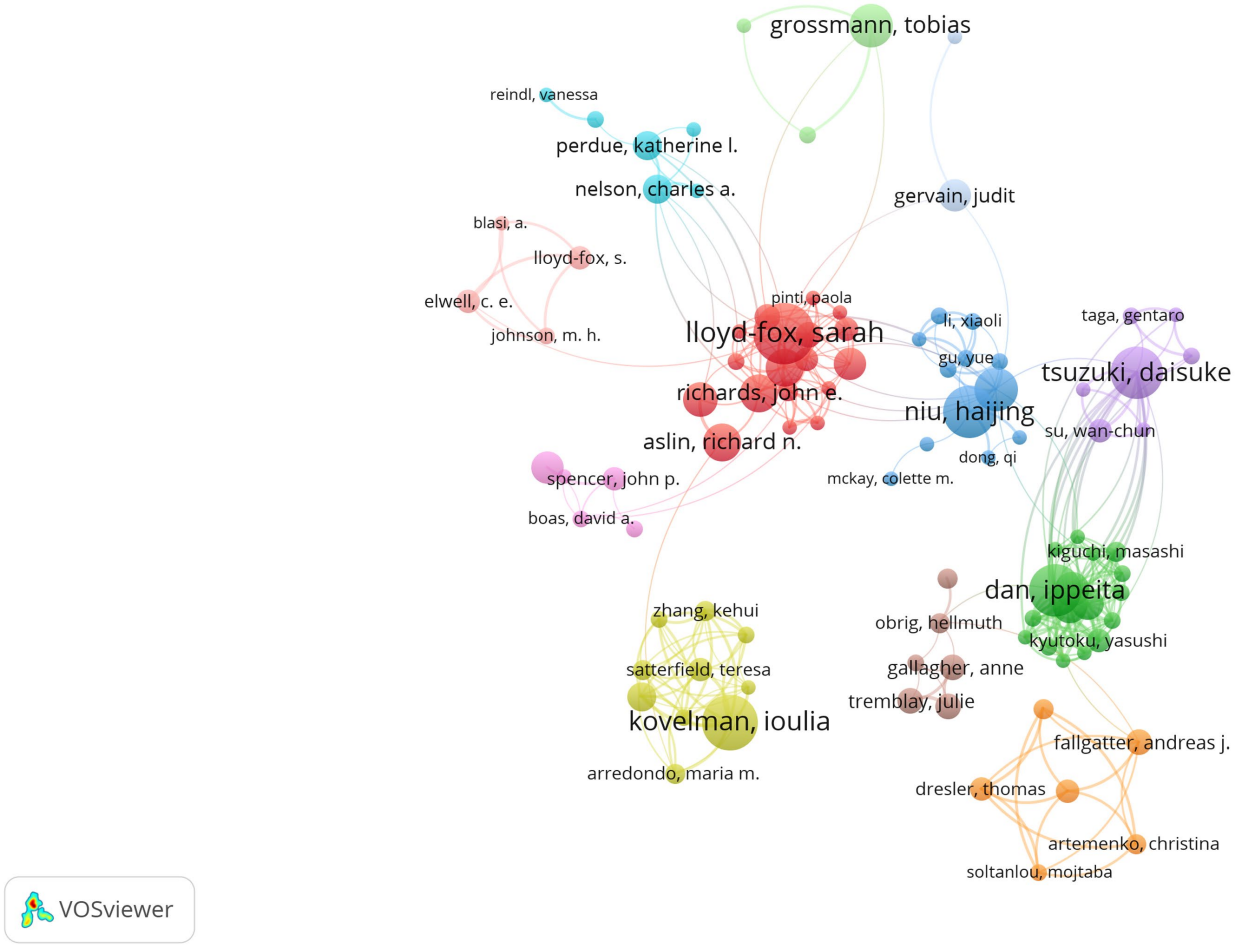
The cluster view of authors.

A total of 2,686 authors have published papers on fNIRS in the field of pediatric research. Table 2 lists the top 10 authors with the most publications. Considering both the number of publications and H-index, Lloyd-Fox Sarah is the most influential author in this field. A collaboration network analysis was conducted for the 147 authors who have published at least 5 papers, revealing that the red nodes cluster represented by Lloyd-Fox Sarah are the most prominent in terms of the number of publications and the breadth of collaborations (Figure 5).

**Table 2:** Publication authors and representative literature.

Rank	Author	Publications	H index	Representative literature	Journal	IF	Citations of literature
1	Lloyd-Fox Sarah	21	14	Near-infrared spectroscopy: a report from the McDonnell infant methodology consortium (29)	Developmental Cognitive Neuroscience	4.6	142
2	Kovelman Ioulia	19	9	Brain basis of phonological awareness for spoken language in children and its disruption in dyslexia (83)	Cerebral Cortex	2.9	74
3	Li Jun	19	8	Narrowband Resting-State fNIRS Functional Connectivity in Autism Spectrum Disorder (61)	Frontiers in Human Neuroscience	2.4	7
4	Dan Ippetita	18	13	Spatial registration for functional near-infrared spectroscopy: from channel position on the scalp to cortical location in individual and group analyses (84)	Neuroimage	4.7	124
5	Niu Haijing	18	10	Resting-state functional brain connectivity: lessons from functional near-infrared spectroscopy (85)	Neuroscientist	3.5	45
6	Tsuzuki Daisuke	18	12	Differences in cortical activation patterns during action observation, action execution, and interpersonal synchrony between children with or without autism spectrum disorder (ASD): An fNIRS pilot study (86)	PLoS One	2.8	17
7	Grossmann Tobias	15	10	Variability in Infants’ Functional Brain Network Connectivity Is Associated With Differences in Affect and Behavior (87)	Frontiers in Psychiatry	3.2	10
8	Minagawa Yasuyo	15	7	Maternal speech shapes the cerebral frontotemporal network in neonates: A hemodynamic functional connectivity study (88)	Developmental Cognitive Neuroscience	4.6	9
9	Perlman Susan B	14	11	Adversity is Linked with Decreased Parent–Child Behavioral and Neural Synchrony (89)	Developmental Cognitive Neuroscience	4.6	7
10	Aslin Richard N	13	9	Comparison of short-channel separation and spatial domain filtering for removal of non-neural components in functional near-infrared spectroscopy signals (90)	Neurophotonics	4.8	20

### 3.4 Analysis of journals

**Figure 6:**
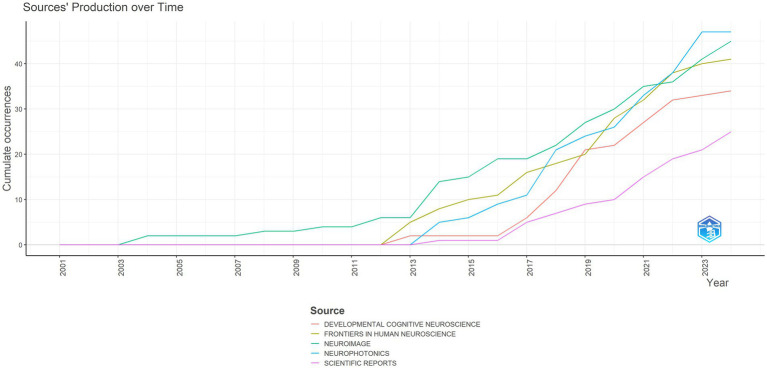
The trend of the number of publications published by the top 5 journals over time.

A total of 239 journals have published papers on fNIRS in the field of pediatric research. Table 3 shows the top 10 journals with the highest number of published papers. “Neurophotonics” has published the most papers, followed by “Neuroimage” and “Frontiers in Human Neuroscience.” In terms of the number of papers and H-index, “Neurophotonics” “Neuroimage” “Frontiers in Human Neuroscience” and “Developmental Cognitive Neuroscience” have a significant impact. Figure 6 displays the trend of the number of papers published by the top 5 journals over time. The publication volume of all five journals shows an increasing trend year by year. As of May 31, 2024, “Neurophotonics” is currently the journal with the highest number of published papers in 2024.

**Table 3:** Ranking of top 10 journal.

Rank	Journal	Publications	H index	IF (2024)
1	Neurophotonics	47	14	5.3
2	Neuroimage	45	25	5.7
3	Frontiers in Human Neuroscience	41	16	2.9
4	Developmental Cognitive Neuroscience	34	16	4.7
5	Scientific Reports	25	9	4.6
6	Frontiers in Neuroscience	24	8	4.3
7	Brain Science	23	5	3.3
8	Developmental Science	22	10	3.7
9	Frontiers in Psychology	19	9	3.8
10	Plos One	19	10	3.7

### 3.5 Analysis of top 10 citations of included records

This study included a total of 27,459 cited references, with Table 4 listing the top 10 most-cited references by the number of citations. The paper with the highest citation rate was published by Huppert et al. in “Applied Optics.” The authors developed a LAB-based graphical user interface program, HomER, and described methods for removing physiological, instrumental, and motion artifact noise from various optical data in fNIRS to facilitate the processing of optical functional brain data (10). Therefore, this paper has been frequently cited as an important reference for scholars processing fNIRS detection data. According to the titles of the cited works listed in Table 4, the top 10 most-cited references mainly cover topics including fNIRS research design, data processing, and other technical and methodological papers.

**Table 4:** Ranking of top 10 cited publications.

Rank	References	Title	Citations
1	Huppert et al. (10). Appl optics	HomER: a review of time-series analysis methods for near-infrared spectroscopy of the brain	195
2	Lloyd-Fox et al. (3). Neurosci Biobehav R	Illuminating the developing brain: the past, present and future of functional near infrared spectroscopy	189
3	Scholkmann et al. (30). Neuroimage	A review on continuous wave functional near-infrared spectroscopy and imaging instrumentation and methodology	92
4	Gervain et al. (29). Dev Cogn Neurosci	Near-infrared spectroscopy: a report from the McDonnell infant methodology consortium	89
5	Ferrari et al. (16). Neuroimage	A brief review on the history of human functional near-infrared spectroscopy (fNIRS) development and fields of application	88
6	Benjamini et al. (31). J R Stat SOC B	Controlling the False Discovery Rate: a Practical and Powerful Approach to Multiple Testing	82
7	Strangman et al. (32). Neuroimage	A quantitative comparison of simultaneous BOLD fMRI and NIRS recordings during functional brain activation	80
8	Delpy et al. (33). Phys Med Biol	Estimation of optical pathlength through tissue from direct time of flight measurement	76
9	Chul et al. (34). Neuroimage	NIRS-SPM: statistical parametric mapping for near-infrared spectroscopy	75
10	Klem et al. (35). Electroencephalogr Clin Neurophysiol Suppl	The ten-twenty electrode system of the International Federation. The International Federation of Clinical Neurophysiology	75

### 3.6 Analysis of keywords

**Figure 7:**
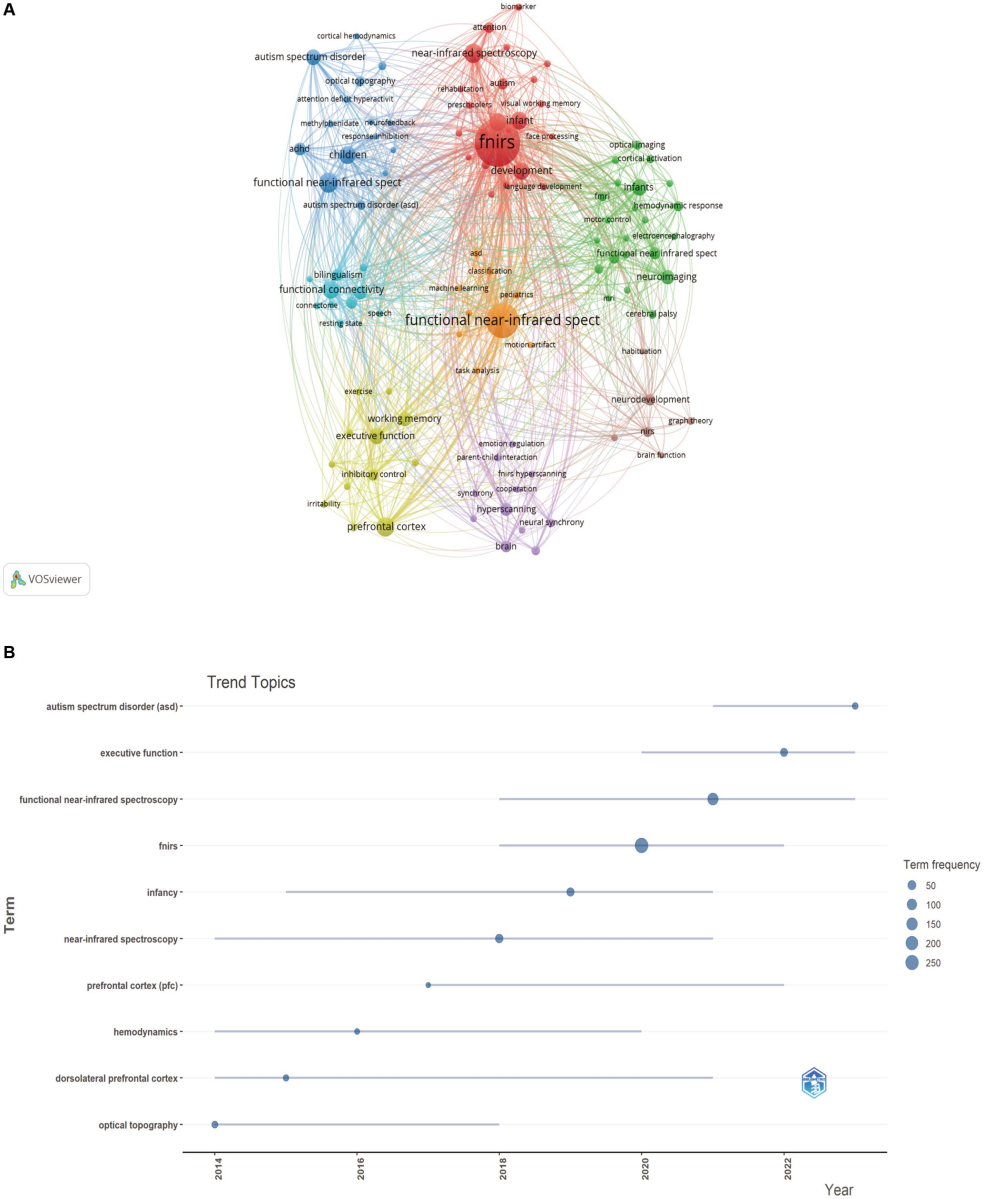
**(A)** The cluster view of high frequency keywords. **(B)** The overlay visualization of the top 10 frequency keywords between 2014 and 2024.

From the 761 publications included in this study, 1,645 keywords were extracted, and network clustering was performed on 109 keywords with a frequency of occurrence greater than or equal to 5, resulting in 8 clusters (Figure 7A). The size of the nodes indicates the frequency of the corresponding keywords. Keywords such as “fNIRS,” “functional near-infrared spectroscopy,” “functional connectivity,” “children,” and “infant” represent the technologies involved and the subjects of research in the field, hence their frequent appearance. Notably, in addition to the above keywords, terms like “autism spectrum disorder” and “ADHD” are prominent in the blue cluster, while “prefrontal cortex,” “executive function,” and “working memory” stand out in the yellow cluster, suggesting that fNIRS research in pediatrics is more extensive in these directions. Figure 7B and Table 5 display the top 10 emergent keywords from 2014 to 2024, indicating the evolution of research hotspots and revealing current research trends and potential future trends. Apart from keywords representing fNIRS technology like “fnirs,” “near-infrared spectroscopy,” and “functional near-infrared spectroscopy,” the keyword with the highest frequency of occurrence in the past decade is “executive function,” with recent and ongoing keywords including “autism spectrum disorder (asd)” and “executive function.”

**Table 5:** The top 10 frequency keywords between 2014 and 2024.

Term	Frequency	Year (Q1)	Year (Median)	Year (Q3)
Optical topography	13	2014	2014	2018
Dorsolateral prefrontal cortex	9	2014	2015	2021
Hemodynamics	6	2014	2016	2020
Prefrontal cortex (pfc)	5	2017	2017	2022
Near-infrared spectroscopy	43	2014	2018	2021
Infancy	37	2015	2019	2021
Fnirs	262	2018	2020	2022
Functional near-infrared spectroscopy	139	2018	2021	2023
Executive function	31	2020	2022	2023
Autism spectrum disorder (asd)	10	2021	2023	2023

## 4 Discussion

Current research on brain function mechanisms is not yet thorough, and the physiological changes in brain function under different behaviors during childhood development, as well as the abnormalities of brain function in pathological states, still need more research to elucidate. fNIRS has advantages in detecting brain functions in children due to its portability, safety, and allowing for subject movement. Especially through fNIRS examination to analyze the abnormal functional states of the brain related to developmental disorders in children, the relevant data may become effective physiological indicators for developmental disorder diseases. This is significant for early identification, diagnosis, assessment, treatment, therapeutic evaluation, and prognosis prediction of such diseases. It can further study the relationship between childhood developmental disorders and brain mechanisms and guide and optimize the design of clinical treatment and rehabilitation programs.

This study conducts a bibliometric analysis of publications on the application of fNIRS in pediatric detection. Prior to this, scholars have already conducted bibliometric studies on the research dynamics within the fNIRS field. Two research teams, led by Wangwang Yan and Devezas, utilized different bibliometric indicators and analysis tools to examine authors, journals, institutions, countries, and networks of co-occurring keywords in the fNIR research domain from 2000 to 2020. They identified infants, social interaction, aging, neural development, cognition, and emotional assessment as research trends (11, 12). Ye et al. unveiled the overview, hotspots, and trends of clinical disease research based on fNIRS from 2011 to 2022, discovering that cutting-edge topics included executive function, functional connectivity, Alzheimer’s disease, children, and adolescents (13). Li et al. analyzed the top 100 most cited articles in the fNIRS field published up to December 31, 2023, using various bibliometric indicators and analysis tools. They statistically profiled these high-impact articles in terms of publishing journals, authors, institutions, etc., and found that near-infrared spectroscopy, activation, cerebral blood flow, brain, neonates, oxygenation, cortex, fMRI, and infants constituted future research directions and potential hotspots in fNIRS (14). Since 2017, the distribution of infant neuroimaging publications has shown an increase in fNIRS research literature (15), indicating a potential shift in infant neuroimaging methods. In light of the research trends revealed by previous bibliometric studies in the fNIRS field, as well as the rapid proliferation of fNIRS technology in pediatric detection, we believe it is essential to further explore the application and development trends of fNIRS technology in pediatric detection. This would provide researchers in the field with a comprehensive perspective, thereby facilitating the determination of research topics and continuing to advance research development in this area.

### 4.1 Principal results

The number of papers published annually is a preliminary indicator for assessing research development. Over the past 20 years, there has been an upward trend in the publication of research papers on fNIRS in the field of pediatrics, with peak periods occurring in 2012 and 2014. The significant increase in publications during these 2 years can be attributed to two main factors. Firstly, the development of fNIRS technology, including the introduction of more channels and the development and application of wearable devices. Since 1999, Hitachi and NIRx have successively released multi-channel commercial fNIRS instruments, gradually increasing temporal resolution and the number of channels (16). Secondly, updates in fNIRS data analysis methods, such as the independent component analysis approach proposed by Han Zhang et al. to reveal resting-state brain functional connectivity (17), and the combination of high-density diffusion optical sensing arrays with optical tomography scanning by Koch et al., which provided high-resolution functional mapping of the human somatosensory cortex (18), offering better analytical forms for more accurate and in-depth interpretation of fNIRS detection data. Moreover, significant progress was made in early explorations of fNIRS in children’s task and resting-state detections. For example, Lloyd Fox et al. found that bilateral posterior temporal regions in five-month-old infants were involved in social brain networks (19), and Carlsson et al. discovered activation in the right frontotemporal cortex when infants recognized their mother’s face (20). These studies not only proved the applicability of fNIRS technology to infants and young children but also laid a foundational basis for fNIRS research in pediatrics. The significant growth in the number of fNIRS applications in pediatric research starting from 2012 was facilitated by instrumentation, data analysis, and foundational early research. Additionally, in 2014, “Neuroimage” published a special issue to commemorate the 20th anniversary of fNIRS technology (21), and the establishment of the fNIRS society that same year, with “Neurophotonics” as its official journal (2), was beneficial to the development of fNIRS research in pediatrics and contributed to the rapid increase in research publications after 2014.

More than 40% of the global fNIRS pediatric research papers come from the United States, with the majority of active research institutions originating from European and American countries, China, and Japan. This is attributed to the development and application of fNIRS technology and equipment in these countries. Looking at the network of institutional and scholarly cooperation, current research primarily focuses on domestic collaborations, while international collaborations are less common. Authors who have made significant contributions and impact in fNIRS pediatric research include Lloyd-Fox Sarah, Kovelman Loulia, and Li Jun. Lloyd-Fox Sarah is among the most prolific and influential scholars in fNIRS pediatric research, dedicating her studies to aspects of fNIRS detection in infant brain development, cognitive functions, motor functions, and social behavior (22–24), which are crucial for exploring the characteristics and mechanisms of infant brain functional development. Kovelman Loulia concentrates on fNIRS research related to language functions in children, such as investigating the neural basis of English language processing and the neural mechanisms of grammar processing in bilingual children (25, 26). Li Jun is committed to fNIRS research on autism spectrum disorders (ASD), using fNIRS technology to explore the pathological features and neural mechanisms of ASD (27, 28).

“Neurophotonics,” as the official journal of the fNIRS Society, is currently the most prolific publisher of fNIRS pediatric research papers, followed by “Neuroimage” and “Frontiers in Human Neuroscience.” Scholars may consider these popular journals for submission. Journals with a high number of publications include those in the fields of imaging, neuroscience, and psychology. Over the past decade, the volume of papers published in these journals has increased year by year, indicating the continuous development of fNIRS pediatric research and its growing popularity. Highly cited common references are particularly helpful for quickly understanding the field, with highly cited literature including reviews on fNIRS technology, research methods, and human applications (3, 10, 16, 29, 30), as well as research papers on fNIRS detection techniques and data analysis methods (31–35).

In the field of bibliometrics, keyword burst analysis can intuitively display frequently occurring keywords over a period of time, which indicate the popularity of these keywords during that period. Over the past decade, scholars have focused on different aspects of fNIRS pediatric research at different times. Early studies mainly included research related to the prefrontal cortex and dorsolateral prefrontal cortex, such as the correlation between prefrontal cortex responses and cognitive functions (36, 37), and the regulation mechanism of the prefrontal cortex during early childhood frustrations (38). Recent hot topics in fNIRS pediatric research are in the areas of neurological mechanisms of executive function and ASD in children.

### 4.2 Application of fNIRS in pediatric research

#### 4.2.1 Scope of clinical research

Researchers conduct brain function studies on subjects of different ages and states to explore mechanisms hidden in neural development, brain characteristics, and disease progression. For infants aged 0–3, fNIRS research focuses on exploring the initial characteristics of the brain and early neural development patterns. For example, Lee used fNIRS to detect the response of 16 sleeping infants to speech stimuli, capturing two simultaneous but independent reaction mechanisms activated at the beginning of stimulation: one is the auditory system’s response to sound stimuli, and the other is a neural inhibition effect induced by the arousal system (39). Another study used fNIRS to explore infants’ cortical responses to dynamic faces and bodies, revealing significant activity in the superior temporal region for both stimulus types. It also identified different developmental trajectories for face and body processing, with older infants showing more extensive responses to faces (40).

For children aged 3–12 in the preschool and school-age periods, the brain develops rapidly, with fast formation of neuronal connections and rapid development of language, emotion, social, cognitive, and motor abilities. Therefore, fNIRS research in this age group focuses on exploring the neural development mechanisms of different functions and the pathological states of related diseases. This includes brain function tests for language (41), executive function (42, 43), motor function (44), social ability (45), and related brain dysfunction diseases such as ASD (46), attention deficit and hyperactive disorder (ADHD) (47, 48), cerebral palsy (CP) (49), etc. Taking fNIRS research on CP as an example, some scholars have studied the differences in brain networks between children with CP and normal children during upper and lower limb movements, finding significant differences in brain network characteristics during movement between children with CP and normal children. These difference indicators can effectively evaluate the real-time impact of motor function and rehabilitation training on brain networks (50, 51), but current research does not support using fNIRS for the diagnosis and clinical classification of CP, and whether there are potential indicators that can predict the onset and future development of CP remains to be explored.

In the adolescent period from 12 to 18 years old, neuronal connections strengthen, cortical thickness increases, and cognition, emotional regulation, social aspects, etc. further develop. For subjects in this age group, fNIRS is used to observe the characteristics of neural function development and its influencing factors (52, 53); in addition, due to changes in hormone levels and high sensitivity of the reward system, adolescents often experience emotional fluctuations and behavioral changes. This period is also when various mental health issues such as anxiety, depression, bipolar disorder, and school phobia first appear, so fNIRS technology is also applied in the study of related pathological mechanisms (54–59).

In the study of pediatric diseases, fNIRS is widely used to capture pathological characteristics of brain activity under disease states. These studies aim to explore diagnostic biomarkers and observe the effects of interventions. Currently, fNIRS in pediatric disease research is mainly used for neurodevelopmental disorders, neural injuries, and diseases caused by mental disorders, including pediatric epilepsy, autism, attention deficit disorder, cerebral palsy, language disorders, depression, etc. (60). The fNIRS technology, with its advantages of being non-invasive, portable, and having high tolerance, demonstrates feasibility in long-term monitoring and therefore has great potential in the diagnosis and evaluation of these diseases. According to the keyword analysis results of this study, recent trends in fNIRS pediatric research have leaned toward autism and attention deficit disorder. This may be because the current understanding of both diseases is still incomplete, and their clinical incidence is increasing, symptoms are complex, and there is a lack of clear biomarkers, making accurate and early diagnosis challenging. For example, Weiting Sun used fNIRS to observe the resting-state brain functional connectivity differences between children with ASD and typically developing children. It was found that within the 0.01–0.02 Hz frequency band, intertemporal lobe functional connectivity was significantly reduced in the ASD group, which could potentially serve as an indicator for predicting autism (61). Meredith Pecukonis used fNIRS to study language development problems in infants at high risk for ASD. The study found that the brain responses during language processing in infants at high risk for autism were different from those at low risk, and this difference was related to later language development (62). Lee’s study compared the differences in prefrontal cortex activity between 14 ADHD patients and 14 typically developing children and found that activation in the right dorsolateral prefrontal area in ADHD patients was significantly different from that in typically developing children (48).

Currently, fNIRS in pediatric research is mainly used to study neural development patterns, brain function characteristics, and pathological features of brain injury, mental disorders, and other diseases. A considerable portion of research has confirmed the applicability of fNIRS in these scenarios and has yielded some beneficial results. However, it is still in the initial stages of research, with diverse experimental paradigms, small sample sizes, and limited research base. More efforts are needed to uncover the potential of fNIRS for clinical diagnosis and monitoring of diseases.

#### 4.2.2 Applications of different spectrometer types

In most cases, fNIRS studies employ Continuous Wave NIRS (CW-NIRS), which only provides relative changes in the concentration of oxygenated and deoxygenated hemoglobin but cannot achieve absolute measurement of these absorbing molecules’ concentrations, thereby introducing a significant source of error (16). Apart from CW-NIRS, there are also Time Domain NIRS (TD-NIRS) and Frequency Domain NIRS (FD-NIRS) illumination types. TD-NIRS uses short pulse lasers to detect photon flight time, offering high precision and spatial resolution advantages. FD-NIRS utilizes a modulated near-infrared light source, then measures the detected light intensity decay and the phase shift of this modulation, experimentally determining estimates of scattering parameters to provide more accurate estimates of tissue scattering characteristics (63). Suemori et al. used TD-NIRS to detect cerebral blood volume in children with congenital heart disease, showing that patients with a single ventricle have higher cerebral blood volume than those with dual ventricles. Moreover, cerebral blood volume is related to factors such as age and central venous pressure, suggesting that TD-NIRS monitoring of cerebral blood volume can better understand the patient’s cerebrovascular dynamics. Several studies have applied FD-NIRS for neonatal cerebral hemodynamic monitoring, including preterm infants (64), neonates with hypoxic–ischemic encephalopathy (65), perioperative monitoring of infants with congenital heart diseases at risk of brain injury (66), and comparing hemodynamic differences between full-term and preterm infants (67). Although TD-NIRS and FD-NIRS can obtain more information to improve detection accuracy, their application in pediatric testing is still limited compared to CW-NIRS due to factors such as larger equipment size, higher cost, fewer channels, and complex operation (68, 69). The technical improvement of both detection methods will be key to promoting their further application in the pediatric field.

#### 4.2.3 Applications of hyperscanning technology

In the field of neuroscience research, traditional studies have focused on the brain activity of individual subjects. However, some scholars have pointed out that the neurobiological basis of human social behavior is also an important but often overlooked topic that requires more attention (70). In recent years, a technique known as hyperscanning has been applied in the field of neuroscience. It can measure the brain activity of two or more interacting subjects simultaneously (71), enabling us to monitor the neural activities related to emotions (72) and cognition (73) between individuals during social interactions. Due to the relatively simple detection method of fNIRS, its application in hyperscanning research has gradually increased over the past decade, especially in fNIRS hyperscanning studies on interactions between infants and caregivers. For example, one study used fNIRS hyperscanning to detect the inter-brain synchrony between 12 children aged 3–5 playing interactively with their mothers, finding increased neural synchrony in the prefrontal cortex and the temporoparietal junction under interactive conditions (74). Additionally, there have been studies investigating the characteristics of brain connectivity in social interactions among individuals with autism (75). These studies not only enrich our understanding of the neural mechanisms of social interaction but also provide new perspectives and methods for research in the field of neuroscience. With further development and application of hyperscanning technology, we hope to achieve more significant discoveries regarding the neurobiological basis of human social behavior.

#### 4.2.4 Application of fNIRS in brain-computer interfaces

Brain-Computer Interfaces (BCIs) utilize signal processing algorithms to extract relevant features from acquired brain signals, decoding intentions or brain states in real time (76). However, most current BCI research primarily focuses on adults, with studies on the applicability of BCIs in children still in their initial stages (77). Due to its portability and the advantage of real-time signal acquisition, fNIRS meets the rapid response requirements of BCIs, and in recent years, there has been some progress in fNIRS-BCI research. Some scholars have attempted fNIRS-BCI research in the pediatric field. Floreani et al. developed a pediatric BCI that uses fNIRS to identify positive and negative emotions based on prefrontal cortex activity. Preliminary studies suggest that fNIRS-BCIs are feasible for recognizing emotions in school-aged children, but validation with larger sample sizes is still needed (78). Erdoğan et al. developed a classification method based on fNIRS signals, using Support Vector Machines (SVM) and Artificial Neural Networks (ANN) to distinguish impulsive adolescents from non-impulsive ones. By analyzing fNIRS signals and various psychological tests, the classification accuracy reached 90%, underscoring the potential of fNIRS combined with machine learning to assist clinical diagnosis (79). In the future, integrating other biosignals (such as EEG) could enhance the spatiotemporal resolution of signals and optimize fNIRS signal processing techniques, potentially further advancing the development of fNIRS-BCI research.

### 4.3 Recommendations for future work

With the continuous advancement of fNIRS technology, including updates to equipment, signal acquisition methods, and analytical approaches, its application scope in pediatric research will gradually expand. Currently, fNIRS pediatric research is primarily limited to cooperation between domestic institutions and authors. To promote the development of this field, it is particularly important to strengthen international collaboration and exchange in the future. Additionally, fNIRS pediatric research involves not only medicine but also disciplines such as computer science and electronic engineering. Therefore, inter-institutional and interdisciplinary cooperation is conducive to ensuring the scientific and reliability of research.

In terms of clinical application, standardized and reproducible studies are essential to achieve the purposes of assisting diagnosis, prognosis judgment, or intervention decision-making, in order to obtain reliable results for support. However, some current studies lack standardized data collection and analysis plans, as well as unified experimental paradigms, testing processes, and analytical methods (80), resulting in their ability to only prove the feasibility of certain research methods (60), and the interpretation of fNIRS results requires more caution (81).

Fortunately, there are already standardized frameworks for fNIRS research papers available for reference (82), which undoubtedly help scholars improve the quality of their papers. In the future, more attention needs to be paid to the standardization of fNIRS research methods to ensure higher intensity research evidence. With technological development, the pursuit of precision and information quantity is increasing, technologies such as FD-NIRS and TD-NIRS may be more widely applied. Meanwhile, fields like hyperscanning and brain-computer interfaces are also worth paying attention to, as their development will open up new possibilities for the application of fNIRS in the pediatric field.

### 4.4 Strengths and limitations

To our knowledge, this study is the first bibliometric analysis of fNIRS research in pediatrics. Two visualization tools were used to analyze collaboration networks, popular journals, and research hotspots, providing scholars with references for understanding research overviews, setting research directions, finding literature, and selecting journals for submission. However, there are limitations to this study; we only used data from WoSCC and included English articles, which may have resulted in a few studies being overlooked. Nevertheless, the data selected based on the inclusion criteria of this study is sufficient to comprehensively understand the current state of fNIRS research in pediatrics and ensure the standardization of the data.

## 5 Conclusion

This study searched and analyzed fINRS pediatric research papers published from the establishment of WoSCC to May 31, 2024. It conducted a comprehensive analysis from aspects such as the number of publications, collaboration relationships, publishing journals, and main authors, providing beneficial reference information for researchers in this field. The study found that the United States is the country with the most publications, University College London is the institution with the most publications, Lloyd-Fox Sarah is the author with the most publications and significant influence, and “Neurophotonics” is the journal with the most publications. Current research hotspots focus on using fNIRS to study executive functions in children and ASD. Moreover, although the analysis results of this study did not directly reflect it, technologies such as hyperscanning and brain-computer interfaces are expected to find more widespread applications in future fNIRS studies with technological advancements. Future efforts should strengthen interdisciplinary and international cooperation and exchanges, jointly contributing to the development of fNIRS technology and the standardization of research.

## Data Availability

The original contributions presented in the study are included in the article/Supplementary material, further inquiries can be directed to the corresponding author.
